# Supersymmetric dark matter after LHC run 1

**DOI:** 10.1140/epjc/s10052-015-3718-9

**Published:** 2015-10-23

**Authors:** E. A. Bagnaschi, O. Buchmueller, R. Cavanaugh, M. Citron, A. De Roeck, M. J. Dolan, J. R. Ellis, H. Flächer, S. Heinemeyer, G. Isidori, S. Malik, D. Martínez Santos, K. A. Olive, K. Sakurai, K. J. de Vries, G. Weiglein

**Affiliations:** DESY, Notkestraße 85, 22607 Hamburg, Germany; High Energy Physics Group, Blackett Laboratory, Imperial College, Prince Consort Road, London, SW7 2AZ UK; Fermi National Accelerator Laboratory, P.O. Box 500, Batavia, IL 60510 USA; Physics Department, University of Illinois at Chicago, Chicago, IL 60607-7059 USA; Physics Department, CERN, 1211 Geneva 23, Switzerland; Antwerp University, 2610 Wilrijk, Belgium; Theory Group, SLAC National Accelerator Laboratory, 2575 Sand Hill Road, Menlo Park, CA 94025-7090 USA; ARC Centre of Excellence for Particle Physics at the Terascale, School of Physics, University of Melbourne, Parkville, 3010 Australia; Theoretical Particle Physics and Cosmology Group, Department of Physics, King’s College London, London, WC2R 2LS UK; H.H. Wills Physics Laboratory, University of Bristol, Tyndall Avenue, Bristol, BS8 1TL UK; Instituto de Física de Cantabria (CSIC-UC), 39005 Santander, Spain; Physik-Institut, Universität Zürich, 8057 Zürich, Switzerland; Universidade de Santiago de Compostela, 15706 Santiago de Compostela, Spain; William I. Fine Theoretical Physics Institute, School of Physics and Astronomy, University of Minnesota, Minneapolis, MN 55455 USA

## Abstract

Different mechanisms operate in various regions of the MSSM parameter space to bring the relic density of the lightest neutralino, $\tilde{\chi }^0_{1}$, assumed here to be the lightest SUSY particle (LSP) and thus the dark matter (DM) particle, into the range allowed by astrophysics and cosmology. These mechanisms include coannihilation with some nearly degenerate next-to-lightest supersymmetric particle such as the lighter stau $\tilde{\tau }_{1}$, stop $\tilde{t}_{1}$ or chargino $\tilde{\chi }^\pm _{1}$, resonant annihilation via direct-channel heavy Higgs bosons *H* / *A*, the light Higgs boson *h* or the *Z* boson, and enhanced annihilation via a larger Higgsino component of the LSP in the focus-point region. These mechanisms typically select lower-dimensional subspaces in MSSM scenarios such as the CMSSM, NUHM1, NUHM2, and pMSSM10. We analyze how future LHC and direct DM searches can complement each other in the exploration of the different DM mechanisms within these scenarios. We find that the ${\tilde{\tau }_1}$ coannihilation regions of the CMSSM, NUHM1, NUHM2 can largely be explored at the LHC via searches for $/ \!\!\!\! E_T$ events and long-lived charged particles, whereas their *H* / *A* funnel, focus-point and $\tilde{\chi }^\pm _{1}$ coannihilation regions can largely be explored by the LZ and Darwin DM direct detection experiments. We find that the dominant DM mechanism in our pMSSM10 analysis is $\tilde{\chi }^\pm _{1}$ coannihilation: parts of its parameter space can be explored by the LHC, and a larger portion by future direct DM searches.

## Introduction

**Table 1:** Summary of the detectability of supersymmetry in the CMSSM, NUHM1, NUHM2, and pMSSM10 models at the LHC in searches for  $/ \!\!\!\! E_T$ events, long-lived charged particles (LL) and heavy *A* / *H* Higgs bosons, and in direct DM search experiments, depending on the dominant mechanism for bringing the DM density into the cosmological range. The symbols $\checkmark$, ($\checkmark$) and $\times$ indicate good prospects, interesting possibilities and poorer prospects, respectively. The symbol – indicates that a DM mechanism is not important for the corresponding model. The LHC information is drawn largely from Figs. 1, 3 and 4, and the direct DM search information from Fig. 8

DM mechanism	Exp’t	Models			
		CMSSM	NUHM1	NUHM2	pMSSM10
${\tilde{\tau }_1}$	LHC	$\checkmark$ $/ \!\!\!\! E_T$ , $\checkmark$ LL	($\checkmark$ $/ \!\!\!\! E_T$ , $\checkmark$ LL)	($\checkmark$ $/ \!\!\!\! E_T$ , $\checkmark$ LL)	($\checkmark$ $/ \!\!\!\! E_T$ ), $\times$ LL
Coann.	DM	($\checkmark$)	($\checkmark$)	$\times$	$\times$
$\tilde{\chi }^\pm _{1}$	LHC	–	$\times$	$\times$	($\checkmark$ $/ \!\!\!\! E_T$ )
Coann.	DM	–	$\checkmark$	$\checkmark$	($\checkmark$)
${\tilde{t}_1}$	LHC	–	–	$\checkmark$ $/ \!\!\!\! E_T$	–
Coann.	DM	–	–	$\times$	–
*A* / *H*	LHC	$\checkmark$ *A* / *H*	($\checkmark$ *A* / *H*)	($\checkmark$ *A* / *H*)	–
Funnel	DM	$\checkmark$	$\checkmark$	($\checkmark$)	–
Focus	LHC	($\checkmark$ $/ \!\!\!\! E_T$ )	–	–	–
Point	DM	$\checkmark$	–	–	–
*h*, *Z*	LHC	–	–	–	($\checkmark$ $/ \!\!\!\! E_T$ )
Funnels	DM	–	–	–	($\checkmark$)

The density of cold dark matter (CDM) in the Universe is now very tightly constrained, in particular by measurements of the cosmic microwave background radiation, which yield $\Omega _\mathrm{CDM} h^2 = 0.1186 \pm 0.0020$ [1] and are consistent with other, less precise, determinations. This determination of the CDM density at the percent level imposes a corresponding constraint on the parameters of any model that provides the dominant fraction of the CDM density. This is, in particular, true for supersymmetric (SUSY) models with conserved *R*-parity in which the CDM is provided by the stable lightest SUSY particle (LSP) [2, 3]. In a series of recent papers incorporating the data from LHC run 1 and elsewhere, we have implemented the dark matter (DM) density constraint in global analyses of the parameter spaces of different variants of the minimal SUSY extension of the Standard Model (MSSM), assuming that the LSP is the lightest neutralino $\tilde{\chi }^0_{1}$. The models studied included the constrained MSSM (CMSSM) with universal soft SUSY-breaking parameters ($m_0, m_{1/2}$ and $A_0$, in standard notation) at the GUT scale [4], the NUHM1(2) in which universality is relaxed for both together (each separately) of the soft SUSY-breaking contributions to the masses-squared of the Higgs multiplets $m_{H_{1,2}}^2$ [4, 5], and a version of the pMSSM10 [6], in which 10 of the effective Lagrangian parameters (3 gaugino masses $M_{1,2,3}$, 2 squark masses $m_{\tilde{q}_{1,2}} \ne m_{\tilde{q}_{3}}$, a common slepton mass $m_{\tilde{\ell }}$, a common trilinear coupling $A_0$, the Higgs mixing parameter $\mu$, the pseudoscalar Higgs mass $M_A$, and the ratio of Higgs vevs $\tan \beta$) are treated as independent inputs specified at the electroweak scale.

Reproducing correctly the cosmological CDM density requires, in general, some special choice of the SUSY model parameters, which may be some particular combination of sparticle masses and/or couplings. Examples of the former include hypersurfaces in the SUSY parameter space where the LSP is almost degenerate in mass with some next-to-lightest SUSY particle (NLSP), such as the lighter stau $\tilde{\tau }_{1}$ [7–14], stop $\tilde{t}_{1}$ [15–21] or chargino $\tilde{\chi }^\pm _{1}$ [22–26], or where $m_{\tilde{\chi }^0_{1}}$ is almost half the mass of a boson such as a heavy Higgs *H* / *A* [27–31], a light Higgs *h* or *Z* [32, 33], in which case rapid direct-channel annihilation may bring the CDM density into the allowed range. Examples of special coupling combinations include the focus-point region [34–39], where the LSP acquires a significant Higgsino component.

We have commented in our previous work on the relevances of these DM mechanisms for our global analyses. Here we discuss systematically which DM mechanisms are dominant in which subspaces of the CMSSM [40–51], NUHM1 [52–55], NUHM2 [54–57], and pMSSM10 (see, for example, [58–72]) parameter spaces, what the corresponding experimental signatures are, and how one might discover SUSY in each of these different DM regions.

Our analysis of the possible detectability of supersymmetry in the CMSSM, NUHM1, NUHM2, and pMSSM10, depending on the dominant DM mechanisms, is summarized in Table 1.

## Measures of mass degeneracy

We first introduce measures on the MSSM parameters that quantify the relevant mass degeneracies and define each of the above-mentioned subspaces in the CMSSM, NUHM1 and NUHM2 [5, 73]:

1 $$\begin{aligned} {\tilde{\tau }_1} \mathrm{~coann.~(pink):} \left( \frac{m_{\tilde{\tau }_1}}{m_{\tilde{\chi }^0_{1}}} - 1 \right)&<0.15, \nonumber \\ \tilde{\chi }^\pm _{1} \mathrm{~coann.~(green):} \left( \frac{m_{\tilde{\chi }^\pm _{1}}}{m_{\tilde{\chi }^0_{1}}} - 1 \right)&\,<\, 0.1, \nonumber \\ {\tilde{t}_1} \mathrm{~coann.~(gray):} \left( \frac{m_{\tilde{t}_1}}{m_{\tilde{\chi }^0_{1}}} \right) - 1&\,<\, 0.2, \nonumber \\ A/H \mathrm{~funnel~(blue):} \left| \frac{M_A}{m_{\tilde{\chi }^0_{1}}} - 2 \right|&\,<\, 0.4, \nonumber \\ \mathrm{Focus~point~(cyan):} \left( \frac{\mu }{m_{\tilde{\chi }^0_{1}}} \right) - 1&\,<\, 0.3. \end{aligned}$$

In each case we also indicate the color coding we use in the subsequent figures. The measures (1) that we use are empirical, but we have verified extensively that CMSSM, NUHM1, and NUHM2 points that satisfy the DM density constraint do fulfill at least one of these conditions, and that they indeed correspond to the dominant DM mechanisms (in the sense of giving the largest fractions of final states, generally ${\gtrsim }50$ %) [5, 73]. We have found that there are some ‘hybrid’ regions where the dominant mechanism requires two of these conditions simultaneously. In particular, there are regions where the dominant DM mechanism is $\tilde{\tau }_1^+ \tilde{\tau }_1^- \rightarrow {\bar{b}} b$ or ${\bar{t}} t$, processes involving both stau coannihilation and annihilation via the *A* / *H* funnel, which we color purple. There are also regions where the chargino coannihilation condition is satisfied simultaneously with the stau coannihilation or *A* / *H* funnel condition. However, a dedicated study using MicrOMEGAs [74] shows that chargino coannihilation is the dominant DM mechanism in these regions, and that hybrid processes dependent on the $m_{\tilde{\chi }^\pm _{1}}$ and some other degeneracy conditions being valid simultaneously are unimportant, so we color these regions the same green as the other chargino coannihilation regions.

The above DM mechanism conditions need to be modified for our analysis of the pMSSM10. First, as we shall see later, funnels due to annihilations via direct-channel *h* and *Z* resonances can be important [32, 33], so for the pMSSM10 we add to (1) the supplementary criteria:

2 $$\begin{aligned} h \mathrm{~funnel~(magenta):} \left| \frac{M_h}{m_{\tilde{\chi }^0_{1}}} - 2 \right|&< 0.4, \nonumber \\ Z \mathrm{~funnel~(orange):} \left| \frac{M_Z}{m_{\tilde{\chi }^0_{1}}} - 2 \right|&< 0.4. \end{aligned}$$

**Fig. 1:**
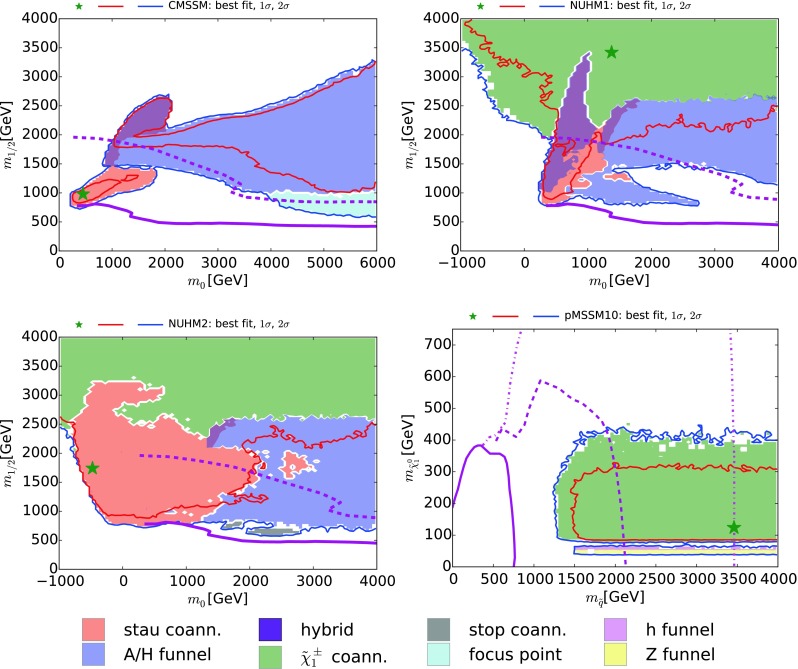
The $(m_0, m_{1/2})$ planes in the CMSSM (*upper left*), the NUHM1 (*upper right*) and the NUHM2 (*lower left*), and the $(m_{\tilde{q}}, m_{\tilde{\chi }^0_{1}})$ plane in the pMSSM10. Regions in which different mechanisms bring the CDM density into the allowed range are *shaded* as described in the legend and discussed in the text. The *red* and *blue* contours are the $\Delta \chi ^2 = 2.30$ and 5.99 contours found in global fits to these models, corresponding approximately to the 68 and 95 % CL contours, with the *green stars* indicating the best-fit points, and the *solid purple* contours show the current LHC 95 % exclusions from  $/ \!\!\!\! E_T$ searches. In the CMSSM, NUHM1, and NUHM2 cases, the *dashed purple* contours show the prospective 5$\sigma$ discovery reaches for  $/ \!\!\!\! E_T$ searches at the LHC with 3000/fb at 14 TeV, corresponding approximately to the 95 % CL exclusion sensitivity with 300/fb at 14 TeV. In the pMSSM10 case, the *dashed purple* contour shows the 95 % CL exclusion sensitivity of the LHC with 3000/fb assuming $m_{\tilde{g}}\gg m_{\tilde{q}}$, and the *dash-dotted lines* bound the corresponding sensitivity region assuming $m_{\tilde{g}}= 4.5 \,\, \mathrm {TeV}$

Second, we find that chargino coannihilation dominates in the pMSSM10 also when the second condition in (1) is relaxed: we use later the condition $|m_{\tilde{\chi }^\pm _{1}}/m_{\tilde{\chi }^0_{1}} - 1| < 0.25$ in our subsequent analysis, which reproduces better the domains of dominance by $\tilde{\chi }^\pm _{1}$ coannihilation.^1^ Finally, we recall that the focusing property of the RGEs is not relevant in the pMSSM10. However, the LSP annihilation rate may still be enhanced when the fifth measure in (1) is satisfied, due to a larger Higgsino component in the LSP, though we find that the dominant DM mechanism in the pMSSM10 generally does not involve this property. We use the same cyan color to identify regions where this condition is satisfied, though it is not due to focus-point behavior.

Our discussion here of DM mechanisms is based on our previously published global likelihood analyses of the CMSSM and NUHM1 [4], the NUHM2 [5] and the pMSSM10 [6]. The reader wishing to know details of our treatments of the various experimental, phenomenological, theoretical and cosmological constraints, as well as our strategies for scanning the parameter spaces of these models is referred to [4–6, 75–77]. However, we note here a couple of important points.

An important contribution to the global $\chi ^2$ function comes from $M_h$, which we evaluate using FeynHiggs 2.10.0 [78]. This combines Feynman-diagrammatic results with a resummation of leading and subleading logarithmic corrections from the stop/top sector. As documented in [78], this is a significant improvement on earlier versions of FeynHiggs, and differences with other earlier codes and their implications are discussed in [79]. FeynHiggs 2.10.0 also provides an estimate of the uncertainty in the calculation of $M_h$. This is in general well approximated by $\pm 1.5 \,\, \mathrm {GeV}$ (thus assigning a $\pm 3 \,\, \mathrm {GeV}$ uncertainty at the 95 % CL), which we use together with the current experimental value of $M_h= 125.09 \pm 0.24 \,\, \mathrm {GeV}$ to calculate the $M_h$ contribution to the global $\chi ^2$ function. Another important contribution to our calculation of the global $\chi ^2$ function is made by the experimental measurement of $(g-2)_\mu$ [80, 81], which differs by ${\sim }3 \sigma$ from many theoretical calculations. This is in tension with LHC constraints on the CMSSM, NUHM1, and NUHM2 [4, 5], but not the pMSSM10 [6].

## Dominant dark matter mechanisms

In this section we discuss the various mechanisms that play dominant rôles in bringing the relic density into the experimentally measured interval in our four models. We display in Fig. 1$(m_0, m_{1/2})$ planes for the CMSSM (upper left) [4], the NUHM1 (upper right) [4] and the NUHM2 (lower left) [5], while for the pMSSM10 we show the $(m_{\tilde{q}}, m_{\tilde{\chi }^0_{1}})$ plane (lower right), where $m_{\tilde{q}}$ denotes the mass of the squarks of the first two generations, which we assume to be common.^2^ The $\Delta \chi ^2 = 2.30$ and 5.99 contours that we found in global fits to these models, corresponding approximately to the 68 and 95 % CL contours, are shown as solid red and blue lines, respectively. Here and elsewhere, the green stars indicate the best-fit points, whose exact locations in some parameter planes are poorly determined and do not carry much useful information, in general, as the $\chi ^2$ minima are quite shallow. Also shown, as solid purple lines, is the current 95 % CL CMSSM exclusion from  $/ \!\!\!\! E_T$ searches at the LHC.^3^ The dashed purple contours in the CMSSM, NUHM1, and NUHM2 cases show the prospective 5$\sigma$ discovery reaches for  $/ \!\!\!\! E_T$ searches at the LHC with 3000/fb at 14 TeV, corresponding approximately to the 95 % CL exclusion sensitivity with 300/fb at 14 TeV. In the pMSSM10 case the dashed purple contour shows the 95 % CL exclusion sensitivity of the LHC with 3000/fb assuming $m_{\tilde{g}}\gg m_{\tilde{q}}$, and the dash-dotted lines bound the corresponding sensitivity region assuming $m_{\tilde{g}}= 4.5 \,\, \mathrm {TeV}$.

All the parameter planes we show are color-coded as listed in (1, 2), with regions where none of these processes are dominant left uncolored. We see in the upper left panel of Fig. 1 that three DM mechanisms dominate in the CMSSM: ${\tilde{\tau }_1}$ coannihilation at low $m_0 \lesssim 2000 \,\, \mathrm {GeV}$, the *H* / *A* funnel at larger $m_0$ and $m_{1/2}$, and the focus point at larger $m_0$ and smaller $m_{1/2}$ where the neutralino becomes a ‘well-tempered’ mixture of bino and Higgsino [82]. There is also a hybrid ${\tilde{\tau }_1}/A/H$ region extending up to $(m_0, m_{1/2}) \sim (2000, 2500) \,\, \mathrm {GeV}$. In the case of the NUHM1 shown in the upper right panel of Fig. 1, there is an analogous ${\tilde{\tau }_1}$ coannihilation region. However, it has a much larger hybrid ${\tilde{\tau }_1}/A/H$ region, which has an extension to low $m_{1/2} \sim 1000 \,\, \mathrm {GeV}$ for $m_0 \lesssim 3000 \,\, \mathrm {GeV}$. On the other hand, $\tilde{\chi }^\pm _{1}$ coannihilation dominates in a large region with $m_{1/2} {\gtrsim }2500 \,\, \mathrm {GeV}$. Here $\mu \ll M_1$, so that the $\tilde{\chi }^0_{1}, \tilde{\chi }^0_{2}$ and $\tilde{\chi }^\pm _{1}$ are nearly degenerate in mass and the $\tilde{\chi }^0_{1}$ has mainly a Higgsino composition. A similar $\tilde{\chi }^\pm _{1}$ coannihilation region is visible in the NUHM2 in the lower left panel of Fig. 1, where we also see a more extensive ${\tilde{\tau }_1}$ coannihilation region extending to $m_0 \sim 2000 \,\, \mathrm {GeV}$,^4^ with a relatively small hybrid ${\tilde{\tau }_1}/A/H$ region. This is the only case where we see a region of dominance by ${\tilde{t}_1}$ coannihilation, in islands around $(m_0, m_{1/2}) \sim (2000, 500) \,\, \mathrm {GeV}$.

In contrast, as shown in the lower right panel of Fig. 1, we found in our version of the pMSSM10 [6] that the dominant DM mechanism is usually $\tilde{\chi }^\pm _{1}$ coannihilation, this time with a Bino-like LSP and $M_1\sim M_2$.^5^ We also note in Fig. 1 bands at low $m_{\tilde{\chi }^0_{1}}$ where rapid annihilations via the *h* and *Z* funnels are dominant. Not shown in Fig. 1 are scatterings of points with $m_{\tilde{\chi }^0_{1}} \gtrsim 300 \,\, \mathrm {GeV}$ where $\tilde{\tau }_1$ coannihilation can also be important, and of points with $m_{\tilde{\chi }^0_{1}} \lesssim 150 \,\, \mathrm {GeV}$ where the fifth condition in (1) comes into play.^6^ We see later that this condition and the $\tilde{\tau }_1$ coannihilation mechanism dominate in specific regions of other projections of the pMSSM10 parameter space, as does ‘bulk’ $\tilde{\chi }^0_{1} \tilde{\chi }^0_{1}$ annihilation where none of the conditions (1) and ( 2) are satisfied.

One may also consider the possibility that the LSP provides only a fraction of the CDM. A complete discussion of this possibility is beyond the scope of this paper, but we note that in some regions, e.g., those dominated by $\tilde{\tau }_1$ or ${\tilde{t}_1}$ coannihilation, lowering the CDM density requires reducing the NLSP–LSP mass difference. This would also have the effect of reducing correspondingly the maximal LSP mass, which would favor sparticle detection at the LHC. On the other hand, direct detection would be more difficult if only a small fraction of the galactic halos were composed of LSPs. For some recent discussions, see [83–85].

Before concluding this section, we note that the marginalization procedure we use to produce the two-dimensional planes we display does not display directly all the physics information that would be contained in a complete set of two-dimensional slices with fixed parameters such as $\tan \beta$ and $A_0$ used in many analyses, cf., [54, 55, 79]. On the other hand, these carry very little if any statistical information about the global parameter spaces. The color coding of the dominant annihilation channels used here contains the information of interest to us in this paper.

## The LHC sensitivity

In this section we discuss the prospective reaches of future LHC searches (see for example, [86, 87]) and their impacts in the contexts of the various preferred DM mechanisms. We will see that in many cases the preferred DM mechanism can be directly probed via the appropriate LHC searches, as summarized in Table 1.

**Fig. 2:**
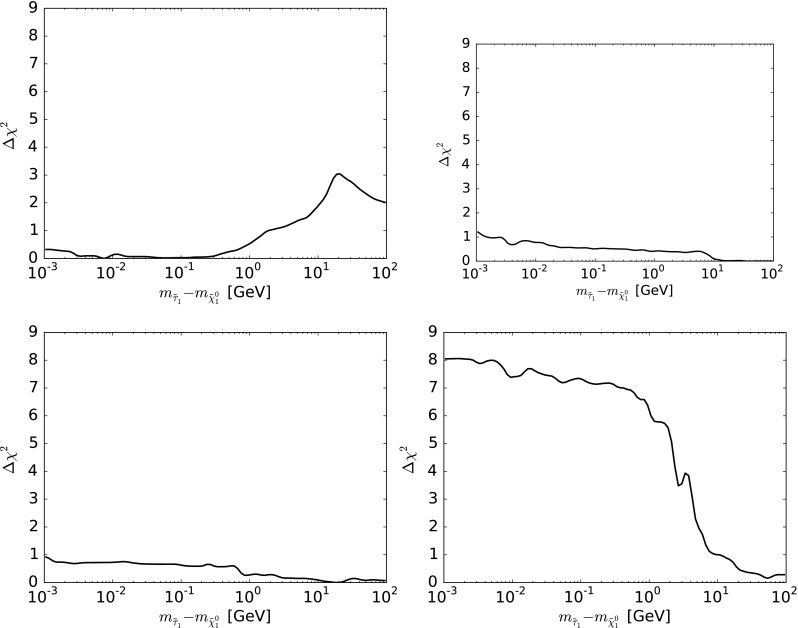
The one-dimensional $\Delta \chi ^2$ profile likelihood functions in the CMSSM (*upper left*), the NUHM1 (*upper right*), the NUHM2 (*lower left*) and the pMSSM10 (*lower right*) for $m_{\tilde{\tau }_1}-m_{\tilde{\chi }^0_{1}} < 100 \,\, \mathrm {GeV}$. In the CMSSM, NUHM1, and NUHM2, low values of $\chi ^2$ are found for points with $m_{\tilde{\tau }_1}- m_{\tilde{\chi }^0_{1}} \sim \,\, \mathrm {MeV}$, whereas in the pMSSM10 $\Delta \chi ^2$ rises to $\sim 8$ at small $m_{\tilde{\tau }_1}- m_{\tilde{\chi }^0_{1}}$

**Fig. 3:**
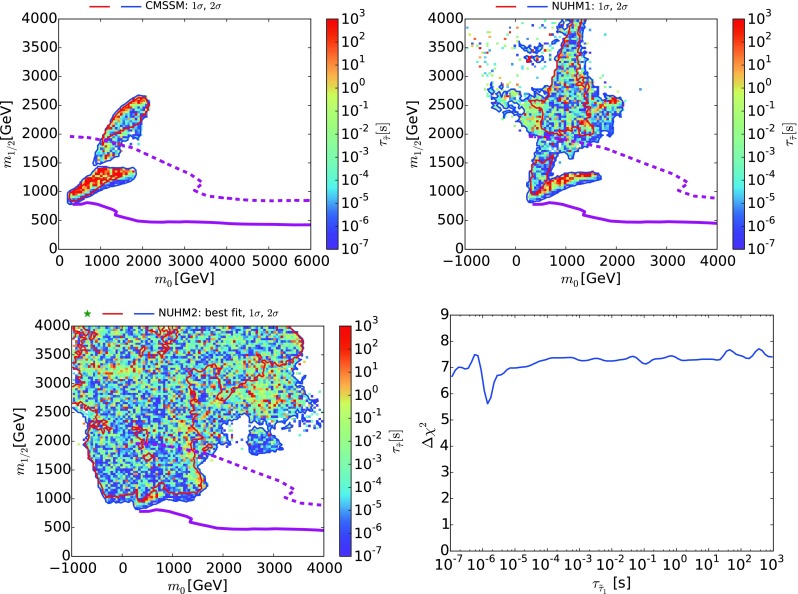
The $(m_0, m_{1/2})$ planes in the CMSSM (*upper left*), the NUHM1 (*upper right*) and the NUHM2 (*lower left*), showing the regions where the lowest-$\chi ^2$ points have $\Delta \chi ^2 < 5.99$ and $10^3$ s $> \tau _{\tilde{\tau }_1} > 10^{-7}$ s: the lifetimes [14] of these points are *color-coded*, as indicated in the legends. The *red* and *blue* contours are for $\Delta \chi ^2 < 2.30 (5.99)$ relative to the absolute minimum. Also shown in these panels as *solid purple* contours are the current LHC 95 % exclusions from  $/ \!\!\!\! E_T$ searches in the ${\tilde{\tau }_1}$ coannihilation regions, and as *dashed purple* contours the prospective 5$\sigma$ discovery reaches for  $/ \!\!\!\! E_T$ searches at the LHC with 3000/fb at 14 TeV, corresponding approximately to the 95 % CL exclusion sensitivity with 300/fb at 14 TeV. As discussed in the text, the sensitivities of LHC searches for metastable ${\tilde{\tau }_1}$’s in the ${\tilde{\tau }_1}$ coannihilation region are expected to be similar [98]. The *lower right panel* shows the one-dimensional $\Delta \chi ^2$ function in the pMSSM10 for the lifetime of the ${\tilde{\tau }_1}$ in the range $10^3$ s $> \tau _\mathrm{NLSP} > 10^{-7}$ s

**Fig. 4:**
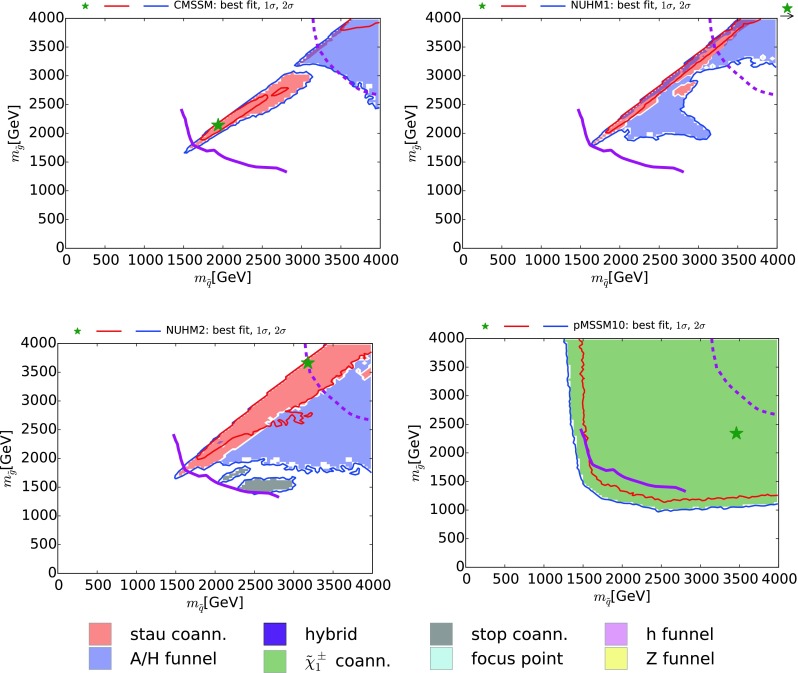
The $(m_{\tilde{q}}, m_{\tilde{g}})$ planes in the CMSSM (*upper left*), the NUHM1 (*upper right*), the NUHM2 (*lower left*) and the pMSSM10 (*lower right*). The *red* and *blue solid lines* are the $\Delta \chi ^2 = 2.30$ and 5.99 contours, and the *solid (dashed) purple lines* are the current and (projected) 95 % exclusion contours for  $/ \!\!\!\! E_T$ searches at the LHC (with 300/fb of data at 14 TeV). The *solid lines* are almost identical with the contours for 5$\sigma$ discovery with 3000/fb

###  searches

Looking now at the physics reach of the LHC with  $/ \!\!\!\! E_T$ searches (we base our analysis on Aad et al. [88, 89]), we see that in the CMSSM the preferred $\tilde{\tau }_1$ stau coannihilation region lies just outside the current LHC 95 % CL exclusion region (solid purple line). On the other hand, the *A* / *H*-funnel region allowed in the CMSSM at the 95 % CL lies well outside this region. However, this is no longer the case in the NUHM1 and particularly the NUHM2, where portions of the *A* / *H*-funnel region lie much closer to the LHC 95 % CL exclusion. This is possible mainly because the Higgs mass constraint is less restrictive in these models. In the CMSSM, the future LHC sensitivity estimated in Fig. 1 of [90] (dashed purple line) covers the region where ${\tilde{\tau }_1}$ coannihilation is dominant, and a part of the hybrid region. It also covers slices of the *H* / *A* funnel region and of the focus-point region. Similar features are seen in the NUHM1, except that the focus-point region is not visible in this case, but the LHC  $/ \!\!\!\! E_T$ search has no sensitivity in the $\tilde{\chi }^\pm _{1}$ coannihilation region. In the case of the NUHM2, the  $/ \!\!\!\! E_T$ search is sensitive to only part of the ${\tilde{\tau }_1}$ coannihilation, and none of the $\tilde{\chi }^\pm _{1}$ coannihilation region, but it does cover part of the *H* / *A* funnel region and all the ${\tilde{t}_1}$ coannihilation region. In the pMSSM10 case, parts of the $\tilde{\chi }^\pm _{1}$ coannihilation region and the low-$m_{\tilde{\chi }^0_{1}}$ band are accessible to future LHC searches,^7^ though the future LHC  $/ \!\!\!\! E_T$ searches are less sensitive if $m_{\tilde{g}}\gg m_{\tilde{q}}$ (dashed line) than if $m_{\tilde{g}}= 4.5 \,\, \mathrm {TeV}$ (dotted line). Table 1 summarizes the observability of $/ \!\!\!\! E_T$ events that we estimate in the different models considered, depending on the dominant DM mechanism in each case.

### The possibility of a long-lived charged sparticle

In some circumstances, a charged sparticle such as the ${\tilde{\tau }_1}$ or the $\tilde{\chi }^\pm _{1}$ may be the NLSP, and be only slightly more massive than the LSP, so that it can in principle be long-lived. As we now show, the presence of a long-lived ${\tilde{\tau }_1}$ is indeed a distinctive prospective signature in the CMSSM, NUHM1, and NUHM2.

Figure 2 displays on log-linear scales the one-dimensional $\Delta \chi ^2$ profile likelihoods as functions of the ${\tilde{\tau }_1} - \tilde{\chi }^0_{1}$ mass differences in the ${\tilde{\tau }_1}$ coannihilation regions in the CMSSM (upper left), the NUHM1 (upper right) and the NUHM2 (lower left). Also shown (in the lower right panel) is the corresponding distribution in the pMSSM10, although in this case the ${\tilde{\tau }_1}$ coannihilation region is disfavored: in this case we see that the likelihood function increases sharply for ${m_{\tilde{\tau }_1}} - m_{\tilde{\chi }^0_{1}} \lesssim 10 \,\, \mathrm {GeV}$, rising to $\Delta \chi ^2 \sim 8$ for very small mass differences.

In the CMSSM, NUHM1, and NUHM2 panels the one-dimensional $\chi ^2$ profile likelihood function is quite a flat function of $m_{\tilde{\tau }_1}- m_{\tilde{\chi }^0_{1}}$, and there are points with $\Delta \chi ^2 \lesssim 1$ that have very small values of this mass difference $\sim \,\, \mathrm {MeV}$. Hence, it is possible that the $\tilde{\tau }_1$ might live long enough ($\tau _{\tilde{\tau }_1} \gtrsim 400$ ns) to appear at the LHC as a long-lived (LL) charged particle, or even long enough ($\tau _{\tilde{\tau }_1} \gtrsim 1000$ s) to affect Big Bang nucleosynthesis [91–97]. However, we emphasize that the mass differences required to realize these possibilities (${\lesssim } 1.2 \,\, \mathrm {GeV}, {\lesssim } 0.1 \,\, \mathrm {GeV}$) require quite special parameter sets. One should presumably require $\tau _{\tilde{\tau }_1} {\lesssim }1000$ s in order to avoid destroying the success of Big Bang nucleosynthesis, a constraint that we impose in the following figures.

If $m_{\tilde{\tau }_1}- m_{\tilde{\chi }^0_{1}} \lesssim 1.2 \,\, \mathrm {GeV}$, corresponding to $\tau _{\tilde{\tau }_1} \gtrsim 400$ ns, the ${\tilde{\tau }_1}$ would live long enough to be detectable at the LHC as a LL charged particle [98]. Figure 3 displays the regions of the $(m_0, m_{1/2})$ plane in the CMSSM (upper left panel), the NUHM1 (upper right panel) and the NUHM2 (lower left panel) where the lowest-$\chi ^2$ point has $10^3$ s $> \tau _{\tilde{\tau }_1} > 10^{-7}$ s: the lifetimes of these points are color-coded, as indicated in the legends. The contours for $\Delta \chi ^2 = 2.30 (5.99)$ relative to the absolute minimum of our data set are shown as solid red and blue lines, respectively.

On the other hand, the lower right panel of Fig. 3 displays the one-dimensional $\Delta \chi ^2$ function in the pMSSM10 for the lifetime of the ${\tilde{\tau }_1}$ in the range $10^3$ s $> \tau _{\tilde{\tau }_1} > 10^{-7}$ s. We see that $\Delta \chi ^2 \gtrsim 6$ throughout the displayed range, indicating that a long-lived ${\tilde{\tau }_1}$ is not expected in the region of the pMSSM10 parameter space that is favored by present data. This is because in our analysis the $(g-2)_\mu$ measurement and the DM constraint favor light sfermions and higgsinos, whereas long-lived charginos typically require sfermion and higgsino masses larger than 10 TeV [99].^8^

**Fig. 5:**
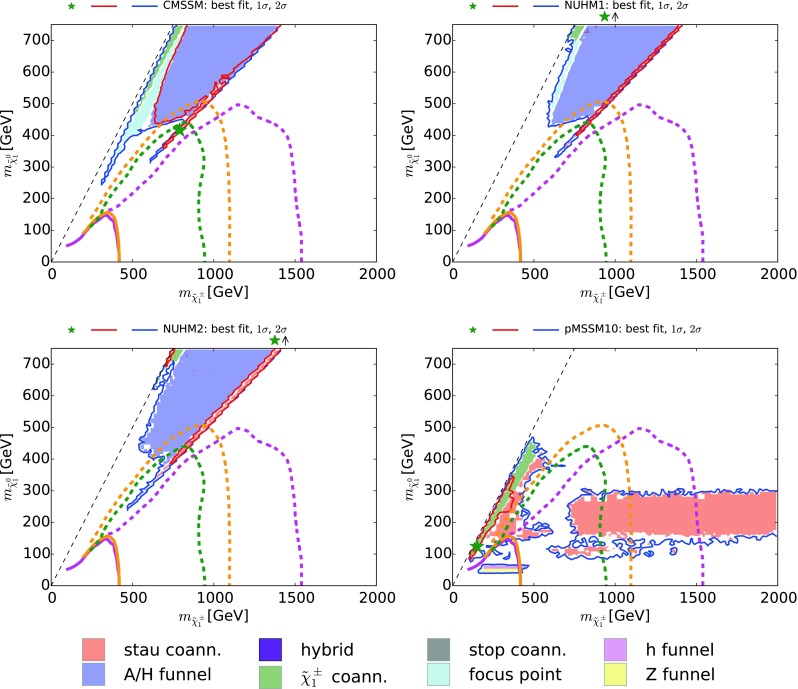
The $(m_{\tilde{\chi }^\pm _{1}}, m_{\tilde{\chi }^0_{1}})$ planes in the CMSSM (*upper left*), the NUHM1 (*upper right*), the NUHM2 (*lower left*) and the pMSSM10 (*lower right*). The *red* and *blue solid lines* are the $\Delta \chi ^2 = 2.30$ and 5.99 contours. The *solid (dashed) orange lines* are the current and projected 3000/fb 95 % CL exclusion sensitivities for $\tilde{\chi }^\pm _{1} \tilde{\chi }^0_{2} \rightarrow W+Z + / \!\!\!\! E_T$ searches [101], the *green dashed lines* the projected 3000/fb 95 % CL exclusion sensitivity for a $\tilde{\chi }^\pm _{1} \tilde{\chi }^0_{2} \rightarrow W+h + / \!\!\!\! E_T$ search [102] (both from [103]), and the *magenta dashed line* is the projected 3000/fb 95 % CL exclusion sensitivity for $\tilde{\chi }^\pm _{1} \tilde{\chi }^0_{2}, \tilde{\chi }^\pm _{1} \tilde{\chi }^\pm _{1} \rightarrow \tau , {\tilde{\tau }} \rightarrow 2, 3 \tau 's + / \!\!\!\! E_T$ searches (from [104])

The sensitivity of the LHC to a long-lived ${\tilde{\tau }_1}$ has been compared in [98] to the sensitivity to  $/ \!\!\!\! E_T$ events, and found to be comparable within the uncertainties. We therefore assume that the projected sensitivity of the LHC to  $/ \!\!\!\! E_T$ events is a good approximation to its sensitivity to parameter sets in the ${\tilde{\tau }_1}$ coannihilation region with $0.1 \,\, \mathrm {GeV}< m_{\tilde{\tau }_1}- m_{\tilde{\chi }^0_{1}} < 1.2 \,\, \mathrm {GeV}$. However, while the reach in the parameter space is similar, the long-lived stau would constitute a spectacular additional signature, and would give complementary information to the direct searches for colored sparticles. The purple contours in the CMSSM, NUHM1, and NUHM2 panels of Fig. 3 again show the present and prospective reaches of the LHC for such events. We infer that long-lived charged particles could be detectable at the LHC throughout the lower parts of the allowed regions in the CMSSM, NUHM1 and NUHM2. Table 1 also summarizes the observability of long-lived charged sparticles in the different models considered.

### Squark and gluino searches

Figure 4 displays the $(m_{\tilde{q}}, m_{\tilde{g}})$ planes for the CMSSM (upper left), the NUHM1 (upper right), the NUHM2 (lower left) and the pMSSM10 (lower right). In each panel we show the $\Delta \chi ^2 = 2.30$ and 5.99 contours as red and blue solid lines, respectively. The current 95 % CL exclusions from ATLAS  $/ \!\!\!\! E_T$ searches are shown as solid purple lines, and the estimated reaches of  $/ \!\!\!\! E_T$ searches for 95 % exclusion with 300/fb of data at 14 TeV [100] (very similar to the reaches for 5$\sigma$ discovery with 3000/fb) are shown as dashed purple lines. The CMSSM panel shows again that the ${\tilde{\tau }_1}$ coannihilation region is within the LHC reach in this model. However, in the NUHM1 and the NUHM2 only portions of the ${\tilde{\tau }_1}$ coannihilation regions are accessible at the LHC, along with small pieces of the *H* / *A* funnel regions. In the case of the NUHM2 the small ${\tilde{t}_1}$ coannihilation regions are also well within the LHC reach.

**Fig. 6:**
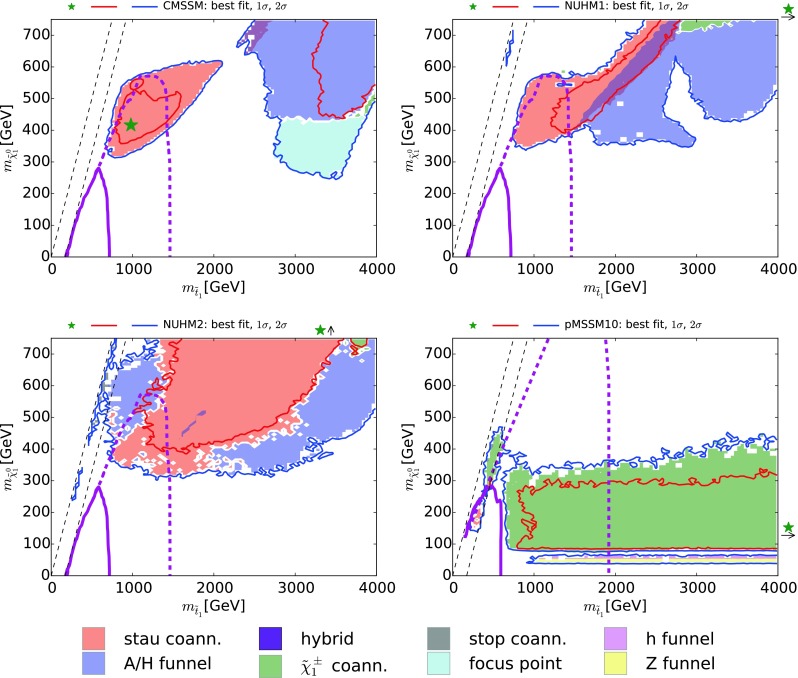
The $(m_{\tilde{t}_1}, m_{\tilde{\chi }^0_{1}})$ planes in the CMSSM (*upper left*), the NUHM1 (*upper right*), the NUHM2 (*lower left*) and the pMSSM10 (*lower right*). The *red* and *blue solid lines* are the $\Delta \chi ^2 = 2.30$ and 5.99 contours. The *diagonal black dashed lines* correspond to $m_{\tilde{t}_1} = m_{\tilde{\chi }^0_{1}}$ and $m_{\tilde{t}_1} = m_t + m_{\tilde{\chi }^0_{1}}$. In each of the CMSSM, NUHM1, and NUHM2 panels, the *solid purple line* is the current 95 % CL limit from the ${\tilde{t}_1} \rightarrow t \tilde{\chi }^0_{1}$ search in [107], and the *dashed purple line* is the 3000/fb projection from [108]. The *solid and dashed purple lines* for the pMSSM10, obtained using [109, 110], respectively, are the LHC run 1 95 % CL limit and the projected 3000/fb 95 % CL exclusion sensitivity with 3000/fb for a ${\tilde{t}_1} \rightarrow b \tilde{\chi }^\pm _{1}$ search, assuming a 100 % branching ratio and $m_{\tilde{\chi }^\pm _{1}} - m_{\tilde{\chi }^0_{1}} = 5 \,\, \mathrm {GeV}$

The pMSSM10 panel shows a completely different picture: $\tilde{\chi }^\pm _{1}$ coannihilation dominates throughout the $(m_{\tilde{q}}, m_{\tilde{g}})$ plane, as discussed at the end of Sect. 3, and the likelihood function is very flat beyond the current LHC  $/ \!\!\!\! E_T$ exclusion. The LHC at 14 TeV will explore a large part of the $(m_{\tilde{q}}, m_{\tilde{g}})$ plane, but a (more) complete exploration would be a task for a higher-energy collider [90].

### Charginos and neutralinos

The differences between the dominant DM mechanisms in the pMSSM10 and the other models studied are highlighted in Fig. 5, which displays the $(m_{\tilde{\chi }^\pm _{1}}, m_{\tilde{\chi }^0_{1}})$ planes in the CMSSM (upper left), the NUHM1 (upper right), the NUHM2 (lower left) and the pMSSM10 (lower right). The diagonal dashed lines indicate where $m_{\tilde{\chi }^\pm _{1}} = m_{\tilde{\chi }^0_{1}}$. As usual, the red and blue solid lines are the $\Delta \chi ^2 = 2.30$ and 5.99 contours. In the pMSSM10 case, the region preferred at the 68 % CL is a narrow strip where $m_{\tilde{\chi }^\pm _{1}} - m_{\tilde{\chi }^0_{1}}$ is small, whereas in the other models much of the 68 % CL region is in a narrow strip where $m_{\tilde{\chi }^\pm _{1}} \sim 2 m_{\tilde{\chi }^0_{1}}$. This reflects the fact that in the CMSSM, NUHM1, and NUHM2 universal boundary conditions are imposed on the gaugino masses at the GUT scale.

We see that ${\tilde{\tau }_1}$ coannihilation dominates over most of the 95 % CL region in this projection of the pMSSM10 parameter space, though not in the 68 % CL region, which has small $m_{\tilde{\chi }^\pm _{1}} - m_{\tilde{\chi }^0_{1}}$ and is where $\tilde{\chi }^\pm _{1}$ coannihilation dominates.^9^ On the other hand, in the CMSSM, NUHM1, and NUHM2, the *H* / *A* funnel dominates most of the 95 % CL regions in the $(m_{\tilde{\chi }^\pm _{1}}, m_{\tilde{\chi }^0_{1}})$ planes, and also the 68 % CL region in the CMSSM, whereas ${\tilde{\tau }_1}$ coannihilation dominates the 68 % CL region in the NUHM1 and the NUHM2, as shown in Fig. 1. At the 95 % CL there are also small $\tilde{\chi }^\pm _{1}$ coannihilation regions in the CMSSM, NUHM1, and NUHM2 where $m_{\tilde{\chi }^\pm _{1}} - m_{\tilde{\chi }^0_{1}}$ is small, and the CMSSM and NUHM1 also have small focus-point regions.

**Fig. 7:**
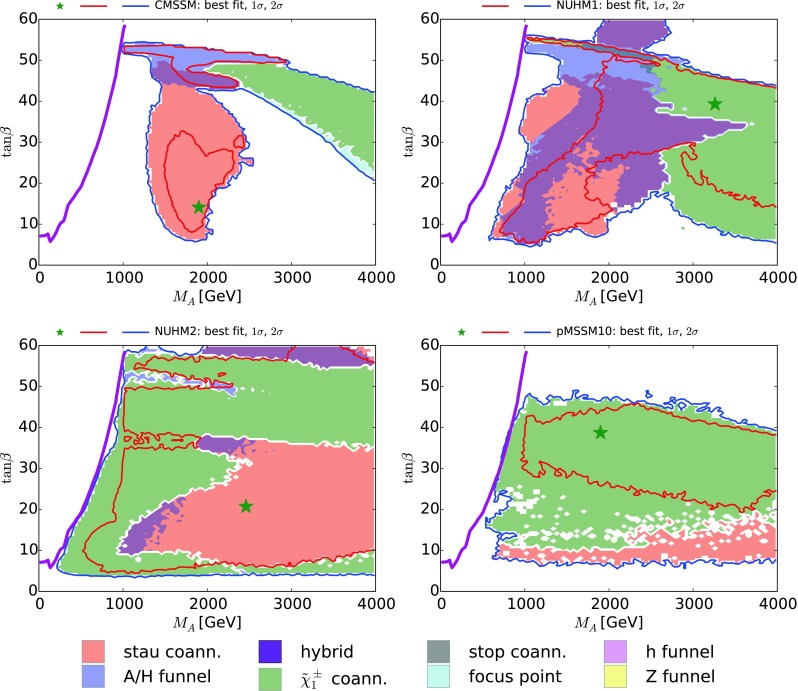
The $(M_A, \tan \beta )$ planes in the CMSSM (*upper left*), the NUHM1 (*upper right*), the NUHM2 (*lower left*) and the pMSSM10 (*lower right*). The *red* and *blue solid lines* are the $\Delta \chi ^2 = 2.30$ and 5.99 contours, and the *solid purple line* is the current LHC 95 % CL exclusion in the $M_h^\mathrm{max}$ scenario

Figure 5 also displays the present and prospective future sensitivities of various LHC searches in the $(m_{\tilde{\chi }^\pm _{1}}, m_{\tilde{\chi }^0_{1}})$ planes. The solid (dashed) orange lines are the current and projected 3000/fb 95 % CL exclusion sensitivities for $\tilde{\chi }^\pm _{1} \tilde{\chi }^0_{2} \rightarrow W+Z + / \!\!\!\! E_T$ searches [101],^10^ the green dashed lines show the projected 3000/fb 95 % CL exclusion sensitivities for the $\tilde{\chi }^\pm _{1} \tilde{\chi }^0_{2} \rightarrow W+h + / \!\!\!\! E_T$ search [102], both taken from [103], and the magenta dashed line is the projected 3000/fb 95 % CL exclusion sensitivity for $\tilde{\chi }^\pm _{1} \tilde{\chi }^0_{2}, \tilde{\chi }^\pm _{1} \tilde{\chi }^\pm _{1} \rightarrow \tau , {\tilde{\tau }} \rightarrow 2, 3 \tau 's + / \!\!\!\! E_T$ searches, taken from [104]. We see that these searches have very limited sensitivities to the CMSSM, NUHM1, and NUHM2, but could explore significant parts of the ${\tilde{\tau }_1}$ coannihilation region in the pMSSM10. However, they would largely miss the $\tilde{\chi }^\pm _{1}$ coannihilation region, i.e., the dominant pMSSM10 DM mechanism cannot be explored by direct searches at the LHC.

### The lighter stop squark

Next we study the differences in the impacts of the dominant DM mechanisms on the pMSSM10 and the other models in the $(m_{\tilde{t}_1}, m_{\tilde{\chi }^0_{1}})$ planes shown in Fig. 6. In each of these planes, we indicate by dashed lines where $m_{\tilde{t}_1} = m_{\tilde{\chi }^0_{1}}$ and where $m_{\tilde{t}_1} = m_t + m_{\tilde{\chi }^0_{1}}$. In the CMSSM, as shown in the upper left panel, we see that the ${\tilde{\tau }_1}$ coannihilation region (which contains all of the parameter space that is allowed at the 68 % CL) is well separated from the *H* / *A* funnel region, and that only a small part of the displayed portion of the $(m_{\tilde{t}_1}, m_{\tilde{\chi }^0_{1}})$ plane is in the hybrid region. In this model we find that $m_{\tilde{t}_{1}} - m_{\tilde{\chi }^0_{1}} \gtrsim 300 \,\, \mathrm {GeV}$ at the 95 % CL, and we do not find a $\tilde{t}_{1}$ coannihilation region, but we do see a focus-point region and a small $\tilde{\chi }^\pm _{1}$ coannihilation region. The situation in the NUHM1 (upper right panel of Fig. 6) exhibits significant differences. The ${\tilde{\tau }_1}$ coannihilation region (which again dominates the 68 % CL region) and the *H* / *A* funnel region still dominate the displayed portion of the $(m_{\tilde{t}_1}, m_{\tilde{\chi }^0_{1}})$ plane, but there is a larger hybrid region, the focus-point region has disappeared and the $\tilde{\chi }^\pm _{1}$ coannihilation region has remained small, but has moved to larger $m_{\tilde{\chi }^0_{1}}$. We also note the appearance of a small $\tilde{t}_{1}$ coannihilation ‘island’ at the 95 % CL in this model. In the case of the NUHM2 (lower left panel), the 68 % CL region is dominated by ${\tilde{\tau }_1}$ coannihilation, whereas its extension to the 95 % CL is dominated by the *H* / *A* funnel, with small areas of $\tilde{\chi }^\pm _{1}$ coannihilation. In this case there is a much more prominent $\tilde{t}_{1}$ coannihilation strip at the 95 % CL. Finally, in the pMSSM10 (lower right panel), we see two patches with small $m_{\tilde{t}_{1}} - m_{\tilde{\chi }^0_{1}}$ connected by a narrow ‘isthmus’, and a ‘continental’ region at large $m_{\tilde{t}_{1}}$, where the 68 % CL is located. As indicated by the green and pink shadings, the dominant DM mechanisms in the ‘islands’ are $\tilde{\chi }^\pm _{1}$ and $\tilde{\tau }_1$ coannihilation, rather than $\tilde{t}_{1}$ coannihilation. We also note the reappearance of the *h* and *Z* funnel bands at low $m_{\tilde{\chi }^0_{1}}$. In this model, the lower-mass ‘island’ and part of the higher-mass ‘island’ can be explored by future LHC searches for ${\tilde{t}_{1}} \rightarrow b \tilde{\chi }^\pm _{1}$ [6], since $m_{\tilde{\chi }^\pm _{1}} \sim m_{\tilde{\chi }^0_{1}}$. This search channel is less powerful in the CMSSM, NUHM1, and NUHM2, where $m_{\tilde{\chi }^\pm _{1}} > 2 m_{\tilde{\chi }^0_{1}}$ in general, particularly in the ${\tilde{\tau }_1}$ coannihilation regions that are favored at the 68 % CL, as seen in Fig. 5.

Figure 6 also displays as purple lines the sensitivities of the most relevant present (solid) and prospective 3000/fb (dashed) searches, namely those for ${\tilde{t}_1} \rightarrow t \tilde{\chi }^0_{1}$ in the CMSSM, NUHM1 and NUHM2 cases, and for $\tilde{t}_{1} \rightarrow b \tilde{\chi }^\pm _{1}$ followed by $\tilde{\chi }^\pm _{1} \rightarrow \tilde{\chi }^0_{1}$ + soft particles in the pMSSM10 case. We see that the current search does not impact the CMSSM, NUHM1 or NUHM2. In the case of the pMSSM10, the solid purple line is the current 95 % CL limit from the $\tilde{t}_{1} \rightarrow b \tilde{\chi }^\pm _{1}$ search in [109], assuming a 100 % branching ratio. This analysis is sensitive to stop topologies with *b* quarks in the final state where the decay products of the subsequent $\tilde{\chi }^\pm _{1}$ decay are undetected, and it was used in [6] to constrain compressed stop spectra. However, it is not sensitive to decays involving on-shell *W* bosons or ${\tilde{t}_1} \rightarrow c \tilde{\chi }^0_{1}$. We conclude that future searches have the potential to explore parts of the ${\tilde{\tau }_1}$ coannihilation regions of the CMSSM, NUHM1, and NUHM2, and of the $\tilde{\chi }^\pm _{1}$ coannihilation region in the pMSSM10 case,^11^ but no DM channel can be fully explored by LHC searches.

### The heavy Higgs bosons

We now study the differences between the dominant DM mechanisms in the pMSSM10 and the other models in the $(M_A, \tan \beta )$ planes shown in Fig. 7. In the case of the CMSSM, the regions allowed at the 95 % CL and preferred at the 68 % CL (blue and red contours, respectively) are generally at considerably larger $M_A$ than the LHC bound (shown as a solid purple line) [111].^12^ The ${\tilde{\tau }_1}$ coannihilation mechanism dominates in a region around $M_A\sim 2000 \,\, \mathrm {GeV}$ for $\tan \beta \lesssim 40$, the *H* / *A* funnel dominates for $\tan \beta \sim 50$, and there is an intermediate hybrid region. On the other hand, $\tilde{\chi }^\pm _{1}$ coannihilation dominates in an arc at larger $M_A$. In the NUHM1, the hybrid and $\tilde{\chi }^\pm _{1}$ coannihilation regions are greatly expanded, and values of $M_A$ closer to the LHC bound are allowed at the 95 % CL. In the NUHM2, on the other hand, essentially all values of $M_A$ consistent with the LHC bound are allowed at the 95 % CL, the $\tilde{\chi }^\pm _{1}$ coannihilation mechanism dominates over most of the $(M_A, \tan \beta )$ plane, leaving ${\tilde{\tau }_1}$ coannihilation to dominate for $M_A\gtrsim 2000 \,\, \mathrm {GeV}$ and $\tan \beta \lesssim 30$. Finally, we see that in the pMSSM10 $\tilde{\chi }^\pm _{1}$ coannihilation dominates the 68 % CL region, that there is also a region at $\tan \beta \lesssim 20$ where ${\tilde{\tau }_1}$ coannihilation may be important, and that there are intermediate uncolored regions where neither of these mechanisms dominate. The LHC bound on $M_A$ is again saturated for $\tan \beta \lesssim 50$.

We have also estimated (not shown) the prospective LHC 95 % CL exclusion sensitivity in the *H* / *A* plane with 300/fb of data for the $m_h^\mathrm{max}$ scenario, scaling the current limit (using results from [114–120]), and comparing with the estimated limits in [121], where good overall agreement was found. We estimate that $M_A\lesssim 2 \,\, \mathrm {TeV}$ could be explored for $\tan \beta \sim 50$, reducing to $M_A\lesssim 1 \,\, \mathrm {TeV}$ for $\tan \beta \sim 20$. This would cover much of the *H* / *A* funnel and hybrid regions in the CMSSM, portions of these regions in the NUHM1, and parts of the $\tilde{\chi }^\pm _{1}$ coannihilation regions in the NUHM2 and the pMSSM10, though not the regions of ${\tilde{\tau }_1}$ coannihilation in these models. Table 1 also summarizes the observability of the heavy Higgs bosons *A* / *H* in the different scenarios considered.

## Direct dark matter searches

**Fig. 8:**
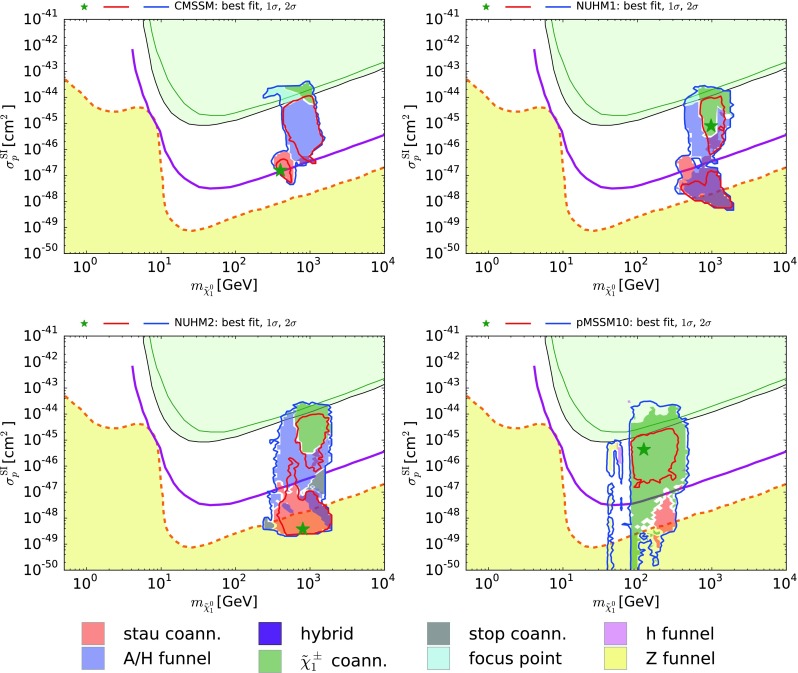
The $(m_{\tilde{\chi }^0_{1}}, \sigma ^\mathrm{SI}_p)$ planes in the CMSSM (*upper left*), the NUHM1 (*upper right*), the NUHM2 (*lower left*) and the pMSSM10 (*lower right*). The *red* and *blue solid lines* are the $\Delta \chi ^2 = 2.30$ and 5.99 contours, and the *solid purple lines* show the projected 95 % exclusion sensitivity of the LUX-Zeplin (LZ) experiment [124]. The *green* and *black lines* show the current sensitivities of the XENON100 [122] and LUX [123] experiments, respectively, and the *dashed orange line* shows the astrophysical neutrino ‘floor’ [126], below which astrophysical neutrino backgrounds dominate (*yellow region*)

We now turn to the capabilities of direct DM search experiments to cast light on the various DM mechanisms. Figure 8 displays the $(m_{\tilde{\chi }^0_{1}}, \sigma ^\mathrm{SI}_p)$ planes for the CMSSM (upper left), the NUHM1 (upper right), the NUHM2 (lower left) and the pMSSM10 (lower right), where $\sigma ^\mathrm{SI}_p$ is the cross section for spin-independent scattering on a proton. Our computation of $\sigma ^\mathrm{SI}_p$ follows the procedure described in [4], and we have once again adopted for the $\pi$-nucleon $\sigma$ term the value $\Sigma _{\pi N} = 50 \pm 7$ MeV. As previously, the $\Delta \chi ^2 = 2.30$ and 5.99 contours are shown as red and blue lines. The sensitivities of the XENON100 [122] and LUX [123] experiments are shown as green and black lines, respectively, and the prospective sensitivity of the LUX-Zeplin (LZ) experiment [124] is shown as a purple line: the projected sensitivity of the XENON1T experiment [125] lies between the current LUX bound and the future LZ sensitivity. Also shown, as a dashed orange line, is the neutrino ‘floor’, below which astrophysical neutrino backgrounds would dominate any DM signal [126] (yellow region).

In the CMSSM case, we see that the current XENON100 and LUX data already put strong pressure on models where the focus-point or $\tilde{\chi }^\pm _{1}$ coannihilation mechanism dominates. There are borderline regions that are formally excluded by the $\sigma ^\mathrm{SI}_p$ data considered in isolation, but become permitted at the 95 % CL in a global fit including other observables, and also due to the uncertainties in the calculation of $\sigma ^\mathrm{SI}_p$ that have been included in the evaluation of the global $\chi ^2$ function [4]. We also see that the $\tilde{\chi }^\pm _{1}$ coannihilation region and most of the *H* / *A* funnel region would be accessible to the planned LZ experiment. However, much of the ${\tilde{\tau }_1}$ coannihilation region lies below the LZ sensitivity, though it could be accessible to a 20-tonne DM experiment such as Darwin [127]. However, this region can be covered in the complementary direct searches at the LHC, as discussed previously. In the case of the NUHM1, the $\tilde{\chi }^\pm _{1}$ coannihilation and *H* / *A* funnel regions still lie largely within the LZ range, and the pure *H* / *A* funnel region is also within reach of LZ. However, the ${\tilde{\tau }_1}$ coannihilation and hybrid regions extend below the reach of LZ and Darwin, even below the neutrino ‘floor’. Again, this region could be partially covered by the complementary LHC direct searches. Similar qualitative conclusions apply to the NUHM2, with the additional observation that the small ${\tilde{t}_1}$ coannihilation regions lie below the LZ sensitivity and straddle the neutrino ‘floor’.

Finally, we see that whereas the region of the pMSSM10 parameter space that is favored at the 68 % CL lies within reach of the LZ experiment, as is the case for much of the $\tilde{\chi }^\pm _{1}$ coannihilation region, there are models in the $\tilde{\chi }^\pm _{1}$ and ${\tilde{\tau }_1}$ coannihilation regions, as well as in the *h* and *Z* funnels and uncolored regions where none of these mechanisms dominate, in which cancellations in the spin-independent matrix element may drive $\sigma ^\mathrm{SI}_p$ below the neutrino ‘floor’. It should be kept in mind here (see the discussion in [6]) that these very low values of $\sigma ^\mathrm{SI}_p$ are due to cancellations (for some discussions of these cancellations, see [128–132]) between different contributions to the matrix element for spin-independent scattering on protons. In [6] it was shown that similar cancellations hold when the cross section for spin-independent scattering on neutrons is considered, instead of the proton case shown in Fig. 8.

Table 1 also summarizes the observability of DM particles in direct searches in the different scenarios considered. We see a degree of complementarity between the LHC and direct DM searches.

We have focused in this article on the prospects for direct searches for DM scattering. A complementary probe of the properties of supersymmetric DM is through indirect detection, searching for the traces of DM annihilation in the Galaxy. A number of recent works have focused on this. For example, [133] has demonstrated that Fermi-LAT satellite limits on $\gamma$-ray emission in dwarf spheroidal galaxies [134] do not currently affect the parameter space of the pMSSM, although they may do so in the future.^13^ Constraints from IceCube and the HESS telescope have been investigated in [136, 137]. IceCube limits [138] do not currently affect the pMSSM10 parameter space, while HESS bounds [139] are primarily on pure wino states, which must have masses greater than a TeV [140, 141] in order to provide a thermal relic. Since the mass of the lightest neutralino is low in our models due to the incorporation of the $(g-2)_{\mu }$ constraint, this does not affect our fits. Accordingly we do not include these data sets in this work, but we plan on implementing likelihoods for these searches in future analyses.

## Summary and conclusions

We have analyzed in this paper the mechanisms that play dominant roles in bringing the relic neutralino density into the range allowed by cosmology in the CMSSM, the NUHM1, the NUHM2 and the pMSSM10. We have delineated the regions of the parameter spaces of these models where dominant roles are played by $\tilde{\tau }_{1}$, $\tilde{t}_{1}$ or $\tilde{\chi }^\pm _{1}$ coannihilation, or funnel regions where the neutralino annihilates rapidly via the heavy Higgs bosons *H* / *A*, and also regions where the neutralino has a significant Higgsino component. In the CMSSM, the NUHM1 and the NUHM2 we find that different mechanisms operate in different regions of the parameter spaces, with relatively small hybrid regions where two mechanisms contribute. In the pMSSM10, $\tilde{\chi }^\pm _{1}$ coannihilation dominates in most of the parameter space, with some contributions from other processes in specific ragions.

Our assessments of the observability of supersymmetry within different models, depending on the dominant DM mechanisms, are summarized in Table 1. Within the CMSSM, the NUHM1 and the NUHM2,  $/ \!\!\!\! E_T$ searches at the LHC can explore significant portions of the $\tilde{\tau }_{1}$ coannihilation regions. These regions also offer the possibility that the $\tilde{\tau }_{1}$ may be relatively long-lived, and detectable at the LHC as a long-lived charged particle. There are regions of the NUHM2 parameter space where $\tilde{t}_{1}$ coannihilation dominates, which can also be explored by  $/ \!\!\!\! E_T$ searches at the LHC. The $\tilde{\chi }^\pm _{1}$ coannihilation and focus-point regions of these models can be explored by the LUX-Zeplin direct DM search experiment. Much of the *H* / *A* funnel regions in these models may be explored via LHC searches for the heavy Higgs bosons, and also via direct DM searches with the LUX-Zeplin and Darwin experiments. On the other hand, the $\tilde{\tau }_{1}$ coannihilation regions seem likely to lie beyond the reaches of the direct DM searches.

Within our analysis of the pMSSM10, $\tilde{\chi }^\pm _{1}$ coannihilation is the dominant DM mechanism in most of the parameter space, though this might change with a different set of independent pMSSM parameters. In addition, there are regions where ${\tilde{\tau }_1}$ coannihilation or direct-channel annihilation via a *h* and *Z* funnel may dominate. Parts of the pMSSM10 model space can be explored at the LHC via  $/ \!\!\!\! E_T$ , *H* / *A* and other searches, and parts by direct DM searches. However, we find no long-lived particle signature in the region of the pMSSM10 parameter space that is currently favored statistically. Overall, large parts of the pMSSM10 parameter space could escape the LHC searches considered here, but a large fraction would be accessible to future DM experiments.

Our analysis shows that the LHC and direct matter searches offer significant prospects for discovering SUSY if it is responsible for the cosmological CDM, and in many cases the mode of discovery can reveal the nature of the dominant mechanism responsible for determining the CDM density. We look forward with interest to learning what the LHC and direct searches will be able to tell us about SUSY DM.

## Footnotes

1 This approach yields results similar to those of [73], where empirical constraints combining the masses and neutralino mixing matrix elements are used.

2 In some cases, these and subsequent figures may include small updates from the versions shown previously [4–6], as they incorporate the latest implementations of the experimental constraints.

3 As discussed in [4, 5], this exclusion curve can be applied to the NUHM1 and NUHM2, also in the range of $m_0 < 0$ shown in these plots, where we interpret negative $m_0 = \mathrm{Sign}(m_0^2) \sqrt{m_0^2}$.

4 Analyses with micrOMEGAs confirm that $\tilde{\chi }^\pm _{1}$ coannihilation processes contribute over 50 % of the final states in most of the green shaded regions for the NUHM1 and NUHM2, and generally over 75 % for $m_{1/2} \gtrsim 3000 \,\, \mathrm {GeV}$. In the NUHM1 and NUHM2 for $m_{1/2} \lesssim 1500 \,\, \mathrm {GeV}$, the $\tilde{\tau }_1$ coannihilation criterion is also satisfied (and that region was colored hybrid in [5]), but $\tilde{\tau }_1$ coannihilation does not contribute significantly to the relic density calculation, so here we color it green.

5 We note, however, that this is the result of an interplay of various constraints, in particular the pMSSM10 assumption of universal slepton masses, as discussed in detail in [6]. A different selection of pMSSM parameters could favor regions with different dominant DM mechanisms, in general. However, this is left for future analysis, and here we simply take over the results of [6].

6 Another micrOMEGAs analysis shows that $\tilde{\chi }^\pm _{1}$ coannihilation processes contribute over 50 % of the final states in most of the green shaded region, and generally over 75 % for $m_{\tilde{\chi }^0_{1}} \lesssim 250 \,\, \mathrm {GeV}$.

7 In principle, these bands are accessible to precise searches for invisible *Z* and *h* decays. However, we found in a survey of the pMSSM10 $(m_{\tilde{q}}, m_{\tilde{\chi }^0_{1}})$ plane in Fig. 1 that the $Z \rightarrow \tilde{\chi }^0_{1} \tilde{\chi }^0_{1}$ branching ratio exceeds the current experimental uncertainty of 1.5 MeV for only a handful of the lowest-$\chi ^2$ points. Likewise, the $h \rightarrow \tilde{\chi }^0_{1} \tilde{\chi }^0_{1}$ branching ratio exceeds 0.1 also for just a handful of points. Invisible *Z* and *h* decays both present opportunities for future searches.

8 The $\Delta \chi ^2$ for a $\tilde{\chi }^\pm _{1}$ in our pMSSM10 sample to have a lifetime in the range $10^3$ s $> \tau _{\tilde{\chi }^\pm _{1}} > 10^{-7}$ s is much greater, so this is an even weaker candidate to be a long-lived charged NLSP.

9 We also note in Fig. 5 the appearance of an uncolored region with $m_{\tilde{\chi }^0_{1}} \lesssim 150 \,\, \mathrm {GeV}$, which is where ‘bulk’ annihilation dominates.

10 These sensitivities assume that the $\tilde{\chi }^\pm _{1}$ and $\tilde{\chi }^0_{2}$ decay exclusively into the $\tilde{\chi }^0_{1}$ in association with *W* and *Z*, respectively, not taking into account the decay $\tilde{\chi }^0_{2} \rightarrow \tilde{\chi }^0_{1} h$ [105, 106].

11 We recall that the *h* and *Z* funnels in the pMSSM10 could in principle be explored by future searches for invisible *h* and *Z* decays.

12 This line was calculated assuming the specific MSSM $m_h^\mathrm{max}$ scenario with $M_\mathrm{SUSY} = 1~\,\, \mathrm {TeV}$ [112, 113], and is used to give a rough impression of the location of the direct heavy Higgs-boson search bounds.

13 Reference [135] appeared while this work was being finalized, and it may be used to constrain the *Z*- and *h*-funnel regions of the pMSSM10. We plan on incorporating this into a future analysis.
